# Brain activity behind the negative and positive emotions: an experimental setting with functional near-infrared spectroscopy (fNIRS)

**DOI:** 10.1192/j.eurpsy.2024.1282

**Published:** 2024-08-27

**Authors:** B. Kabella, A. Fehér, J. Lazáry

**Affiliations:** ^1^Psychiatry, National Institute of Mental Health- Neurology and Neurosurgery; ^2^Semmelweis University – Medicine and Health Sciences, Budapest, Hungary

## Abstract

**Introduction:**

The fNIRS is an optical brain monitoring technique which uses near-infrared spectroscopy for the purpose of functional neuroimaging. Using fNIRS, brain activity is measured by using near-infrared light to estimate cortical hemodynamic activity which occurs in response to neural activity. In the aspect of psychiatry fNIRS is a tool that can potentially facilitate the clinical diagnostic process and identify stages of psychiatric illnesses by providing objective and quantifiable evidences of brain changes. However, this will require specific cerebral haemodynamic patterns to be validated in larger clinical populations with specific psychiatric disorders.

**Objectives:**

Our team decided to set a fNIRS system to find out the difference in prefrontocortical (PFC) activity pattern between healthy and anhedonic population. This abstract has been created for introduce our first findings about the difference in PFC activity under emotionally positively or negatively coloured stimuli in healthy population.

**Methods:**

We have measured 5 healthy adults, non-anhedonic participants under emotionally different visual and acustic stimuli with the use of NIRX/NIRScout system with the view of our prefronto-temporo-parietal montege.

**Fig 1** We devided our experimental tools into 4 individual 20 second long parts:

Passive neutral visual or acustic stimuli for baseline (watching a black dot or scilence)

Passive visual stimuli (watching a single picture)

Active visual stimuli (choosing from photo collage) to detect contrast of cortical background activation

Passive acustic stimuli (listening sounds)

In total, we defined emotionally, two neutral, four positive and four negative stimuli in our experimental setting.

Softwares: We used *HOMER3* for analyzing our datas and estimate hemodinamic reponse factor (HRF) using general linear matrix (GLM) regression. We used *AtlasViewer* for reconstructing HRF image onto the Colin27 digitalised brain model. And we also used *SPSS* for statictical analysis between stimuli types and HRF means.

**Results:**

**Fig 2** Significant HRF differences were measured in the dominant hemisphere dorsolateral prefrontal cortex (DLPFC) between the influence of each emotionally negative and positive stimuli (p<0.001). The level of DLPFC activity was positively influenced by emotionally positive stimuli (p<0.001).

**Image:**

**Figure:**
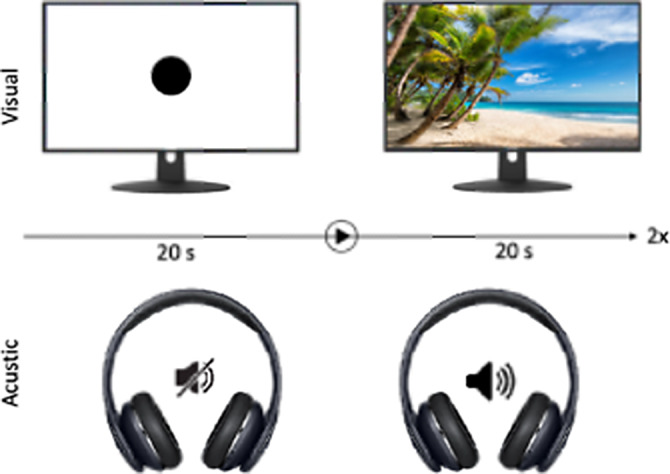

**Image 2:**

**Figure:**
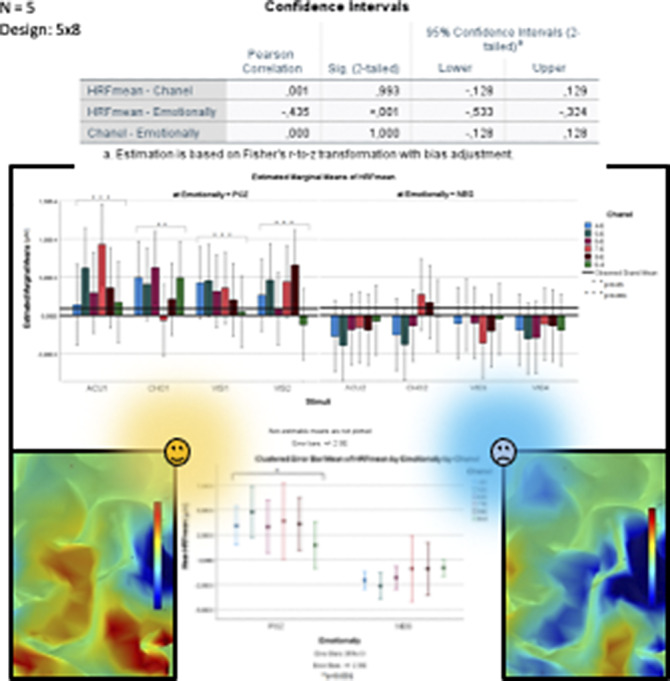

**Image 3:**

**Figure:**
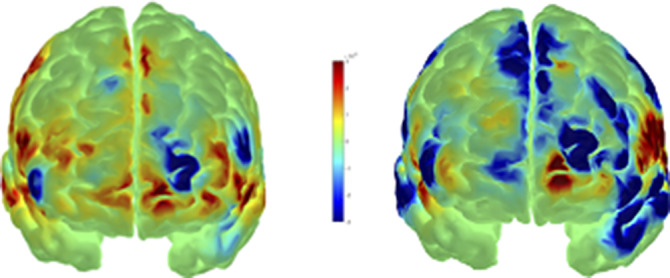

**Conclusions:**

**Fig 3** Our fNIRS experimental system is a suitable tool to measure and model the pattern of prefrontal cortical activity.

Based on the measured hemodynamic values, we detected a significant activity difference in the dominant hemisphere DLPFC during emotionally positive and negative stimuli, the extent of which is positively influenced by emotionally positive stimuli.

The left DLPFC appears to be a promising target for our next studies of anhedonia.

**Disclosure of Interest:**

None Declared

